# Riesz potential and its commutators on Orlicz spaces

**DOI:** 10.1186/s13660-017-1349-4

**Published:** 2017-04-13

**Authors:** Vagif S Guliyev, Fatih Deringoz, Sabir G Hasanov

**Affiliations:** 1 Department of Mathematics, Ahi Evran University, Kirsehir, Turkey; 2 S.M. Nikol’skii Institute of Mathematics, RUDN University, 6 Miklukho-Maklay St, Moscow, 117198 Russia; 3 Ganja State University, Ganja, Azerbaijan

**Keywords:** 32A37, 42B25, 42B35, 46E30, 47B47, Orlicz space, Riesz potential, commutator, BMO, Lipschitz space

## Abstract

In the present paper, we shall give necessary and sufficient conditions for the strong and weak boundedness of the Riesz potential operator $I_{\alpha}$ on Orlicz spaces. Cianchi (J. Lond. Math. Soc. 60(1):247-286, 2011) found necessary and sufficient conditions on general Young functions Φ and Ψ ensuring that this operator is of weak or strong type from $L^{\Phi}$ into $L^{\Psi}$. Our characterizations for the boundedness of the above-mentioned operator are different from the ones in (Cianchi in J. Lond. Math. Soc. 60(1):247-286, 2011). As an application of these results, we consider the boundedness of the commutators of Riesz potential operator $[b,I_{\alpha }]$ on Orlicz spaces when *b* belongs to the BMO and Lipschitz spaces, respectively.

## Introduction

Norm inequalities for several classical operators of harmonic analysis have been widely studied in the context of Orlicz spaces. It is well known that many of such operators fail to have continuity properties when they act between certain Lebesgue spaces and, in some situations, the Orlicz spaces appear as adequate substitutes.

The Hardy-Littlewood maximal operator *M* and the Riesz potential operator $I_{\alpha }$ ($0<\alpha <n$) are defined by

$$M f(x)=\sup_{t>0} \bigl\vert B(x,t) \bigr\vert ^{-1} \int_{B(x,t)} \bigl\vert f(y) \bigr\vert \,dy,\qquad I_{\alpha } f(x)= \int_{{\mathbb{R}^{n}}} \frac{f(y)}{ \vert x-y \vert ^{n-\alpha }}\,dy. $$

Here and everywhere in the sequel $B(x,r)$ is the ball in ${\mathbb {R}^{n}}$ of radius *r* centered at *x* and $\vert B(x,r) \vert =v_{n} r^{n}$ is its Lebesgue measure, where $v_{n}$ is the volume of the unit ball in ${\mathbb{R}^{n}}$.

The commutators generated by a suitable function *b* and the operator $I_{\alpha }$ are formally defined by

$$ [b,I_{\alpha }]f=I_{\alpha }(bf)-bI_{\alpha }(f), $$

respectively.

Given a measurable function *b* the operators $M_{b}$ and $\vert b,I_{\alpha } \vert $ are defined by

$$ M_{b}(f) (x)=\sup_{t>0} \bigl\vert B(x,t) \bigr\vert ^{-1} \int_{B(x,t)} \bigl\vert b(x)-b(y) \bigr\vert \bigl\vert f(y) \bigr\vert \,dy $$

and

$$ \vert b,I_{\alpha } \vert f(x)= \int_{{\mathbb{R}^{n}}}\frac{ \vert b(x)-b(y) \vert }{ \vert x-y \vert ^{n-\alpha }}f(y)\,dy. $$

In [1], Cianchi found necessary and sufficient conditions on general Young functions Φ and Ψ ensuring that the operator $I_{\alpha }$ is of weak or strong type from $L^{\Phi}$ into $L^{\Psi}$. Another boundedness statement with only sufficient conditions for the operator $I_{\alpha }$ on Orlicz spaces was given by Nakai [2]. Note that in [2] a more general case of generalized fractional integrals was studied. Commutators of classical operators of harmonic analysis play an important role in various topics of analysis and PDE; see for instance [3, 4], and the references therein. In [5], Fu *et al.* gave the sufficient conditions for the boundedness of the commutator $[b,I_{\alpha }]$ on Orlicz spaces.

The main purpose of this paper is to give characterizations for the strong and weak boundedness of the Riesz potential on Orlicz spaces. Our characterizations for the boundedness of the operator $I_{\alpha }$ are different from the ones in [1]. As an application of these results, we consider the boundedness of the commutators of Riesz potential operator on Orlicz spaces when *b* belongs to the BMO and Lipschitz spaces, respectively.

We use the notation $A \lesssim B$, which means that $A \le C B$ with some positive constant *C* independent of appropriate quantities. If $A \lesssim B$ and $B \lesssim A$, we write $A\approx B$ and say that *A* and *B* are equivalent.

## Preliminaries; on Young functions and Orlicz spaces

We recall the definition of Young functions.

### Definition 2.1

A function $\Phi: [0,\infty) \rightarrow[0,\infty]$ is called a Young function if Φ is convex and left-continuous, $\lim_{r\rightarrow+0} \Phi(r) = \Phi (0) = 0$ and $\lim_{r\rightarrow\infty} \Phi(r) = \infty$.

From the convexity and $\Phi(0) = 0$ it follows that any Young function is increasing.

The set of Young functions such that

$$ 0< \Phi(r)< \infty\quad\text{for } 0< r< \infty $$

is denoted by $\mathcal{Y}$. If $\Phi\in\mathcal{Y}$, then Φ is absolutely continuous on every closed interval in $[0,\infty)$ and bijective from $[0,\infty)$ to itself.

For a Young function Φ and $0 \leq s \leq\infty$, let

$$\Phi^{-1}(s)=\inf\bigl\{ r\geq0: \Phi(r)>s\bigr\} . $$

If $\Phi\in\mathcal{Y}$, then $\Phi^{-1}$ is the usual inverse function of Φ.

It is well known that

2.1 $$ r\leq\Phi^{-1}(r)\widetilde{\Phi}^{-1}(r)\leq2r\quad \text{for } r\geq0, $$

where $\widetilde{\Phi}(r)$ is defined by

$$ \widetilde{\Phi}(r)= \textstyle\begin{cases} \sup\{rs-\Phi(s): s\in[0,\infty)\} , & r\in[0,\infty), \\ \infty,& r=\infty. \end{cases} $$

A Young function Φ is said to satisfy the $\Delta_{2}$-condition, denoted also as $\Phi\in\Delta_{2}$, if

$$\Phi(2r)\le C\Phi(r),\quad r>0, $$

for some $C>1$. If $\Phi\in\Delta_{2}$, then $\Phi\in\mathcal {Y}$. A Young function Φ is said to satisfy the $\nabla _{2}$-condition, denoted also by $\Phi\in\nabla_{2}$, if

$$\Phi(r)\leq\frac{1}{2C}\Phi(Cr),\quad r\geq0, $$

for some $C>1$.

### Definition 2.2

Orlicz space

For a Young function Φ, the set

$$L^{\Phi}\bigl({\mathbb{R}^{n}}\bigr)= \biggl\{ f\in L^{1}_{\mathrm{loc}}\bigl({\mathbb{R}^{n}}\bigr): \int_{{\mathbb{R}^{n}}}\Phi \bigl(k \bigl\vert f(x) \bigr\vert \bigr)\,dx< \infty \text{ for some }k>0 \biggr\} $$

is called Orlicz space. If $\Phi(r)=r^{p}, 1\le p<\infty$, then $L^{\Phi}({\mathbb{R}^{n}})=L^{p}({\mathbb{R}^{n}})$. If $\Phi(r)=0, (0\le r\le1)$ and $\Phi (r)=\infty, (r> 1)$, then $L^{\Phi}({\mathbb{R}^{n}})=L^{\infty}({\mathbb{R}^{n}})$. The space $L^{\Phi}_{\mathrm{loc}}({\mathbb{R}^{n}})$ is defined as the set of all functions *f* such that $f\chi_{B}\in L^{\Phi}({\mathbb{R}^{n}})$ for all balls $B \subset {\mathbb{R}^{n}}$.

$L^{\Phi}({\mathbb{R}^{n}})$ is a Banach space with respect to the norm

$$\Vert f \Vert _{L^{\Phi}}=\inf \biggl\{ \lambda>0: \int_{{\mathbb{R}^{n}}}\Phi \biggl(\frac { \vert f(x) \vert }{\lambda} \biggr)\,dx\leq1 \biggr\} . $$

For a measurable set $\Omega\subset\mathbb{R}^{n}$, a measurable function *f* and $t>0$, let $m(\Omega, f, t)= \vert \{x\in\Omega: \vert f(x) \vert >t\} \vert $. In the case $\Omega=\mathbb{R}^{n}$, we for brevity denote it by $m(f, t)$.

### Definition 2.3

The weak Orlicz space

$$WL^{\Phi}\bigl(\mathbb{R}^{n}\bigr)=\bigl\{ f\in L^{1}_{\mathrm{loc}}\bigl(\mathbb {R}^{n}\bigr):\Vert f \Vert_{WL^{\Phi}}< \infty\bigr\} $$

is defined by the norm

$$\Vert f\Vert_{WL^{\Phi}}=\inf \biggl\{ \lambda>0 : \sup_{t>0} \Phi (t)m \biggl(\frac{f}{\lambda}, t \biggr) \leq1 \biggr\} . $$

We note that $\Vert f\Vert_{WL^{\Phi}}\leq\Vert f\Vert_{L^{\Phi}}$,

$$\sup_{t>0}\Phi(t)m(\Omega, f, t)=\sup_{t>0}t m\bigl(\Omega, f, \Phi ^{-1}(t)\bigr)= \sup_{t>0}t m \bigl(\Omega, \Phi\bigl( \vert f \vert \bigr), t\bigr) $$

and

2.2 $$ \int_{\Omega}\Phi \biggl(\frac{ \vert f(x) \vert }{ \Vert f \Vert _{L^{\Phi}(\Omega )}} \biggr)\,dx\leq1, \qquad \sup _{t>0}\Phi(t)m \biggl(\Omega, \frac{f}{ \Vert f \Vert _{WL^{\Phi}(\Omega)}}, t \biggr) \leq1, $$

where $\Vert f \Vert _{L^{\Phi}(\Omega)}= \Vert f\chi_{\Omega} \Vert _{L^{\Phi}}$ and $\Vert f \Vert _{WL^{\Phi}(\Omega)}= \Vert f\chi_{\Omega} \Vert _{WL^{\Phi}}$.

The following analogue of the Hölder inequality is well known (see, for example, [6]).

### Theorem 2.4

*Let* $\Omega\subset{\mathbb{R}^{n}}$ *be a measurable set and functions* *f* *and* *g* *measurable on* Ω. *For a Young function* Φ *and its complementary function* Φ̃, *the following inequality is valid*:

$$\int_{\Omega} \bigl\vert f(x)g(x) \bigr\vert \,dx \leq2 \Vert f \Vert _{L^{\Phi}(\Omega)} \Vert g \Vert _{L^{\widetilde{\Phi}}(\Omega)}. $$

By elementary calculations we have the following property.

### Lemma 2.5

*Let* Φ *be a Young function and* *B* *be a set in* $\mathbb{R}^{n}$ *with finite Lebesgue measure*. *Then*

$$ \Vert \chi_{B} \Vert _{L^{\Phi}} = \Vert \chi_{B} \Vert _{WL^{\Phi}}=\frac{1}{\Phi ^{-1} ( \vert B \vert ^{-1} )}. $$

By Theorem 2.4, Lemma 2.5 and (2.1) we get the following estimate.

### Lemma 2.6

*For a Young function* Φ *and* $B=B(x,r)$, *the following inequality is valid*:

$$\int_{B} \bigl\vert f(y) \bigr\vert \,dy \leq2 \vert B \vert \Phi^{-1} \bigl( \vert B \vert ^{-1} \bigr) \Vert f \Vert _{L^{\Phi}(B)}. $$

In the next section, where we prove our main estimates, we use the following theorem.

### Theorem 2.7

[7]

*Let* Φ *be a Young function*.

(i) *The operator* *M* *is bounded from* $L^{\Phi}({\mathbb{R}^{n}})$ *to* $WL^{\Phi }({\mathbb{R}^{n}})$, *and the inequality*
  2.3 $$ \Vert M f \Vert _{WL^{\Phi}}\leq C_{0} \Vert f \Vert _{L^{\Phi}} $$
  *holds with constant* $C_{0}$ *independent of* *f*.
(ii) *The operator* *M* *is bounded on* $L^{\Phi}({\mathbb{R}^{n}})$, *and the inequality*
  2.4 $$ \Vert M f \Vert _{L^{\Phi}}\leq C_{0} \Vert f \Vert _{L^{\Phi}} $$
  *holds with constant* $C_{0}$ *independent of* *f* *if and only if* $\Phi\in \nabla_{2}$.

## Riesz potential in Orlicz spaces

In this section we find necessary and sufficient conditions for the strong/weak boundedness of the Riesz potential operator on Orlicz spaces.

We recall that, for functions Φ and Ψ from $[0,\infty)$ into $[0,\infty]$, the function Ψ is said to dominate Φ globally if there exists a positive constant *c* such that $\Phi(s)\le\Psi(cs)$ for all $s\geq0$.

In the theorem below we also use the notation

3.1 $$ \widetilde{\Psi}_{P}(s)= \int_{0}^{s}r^{P^{\prime}-1}\bigl(\mathcal {B}_{P}^{-1}\bigl(r^{P^{\prime}}\bigr)\bigr)^{P^{\prime}}\,dr, $$

where $1< P\le\infty$ and $\widetilde{\Psi}_{P}(s)$ is the Young conjugate function to $\Psi_{P}(s)$, and

3.2 $$ \Phi_{P}(s)= \int_{0}^{s}r^{P^{\prime}-1}\bigl(\mathcal {A}_{P}^{-1}\bigl(r^{P'}\bigr)\bigr)^{P'}\,dr, $$

where $\mathcal{B}_{P}^{-1}(s)$ and $\mathcal{A}_{P}^{-1}(s)$ are inverses to

$$\mathcal{B}_{P}(s)= \int_{0}^{s}\frac{\Psi(t)}{t^{1+P^{\prime}}}\,dt \quad\text{and} \quad \mathcal{A}_{P}(s)= \int_{0}^{s}\frac{\widetilde{\Phi }(t)}{t^{1+P^{\prime}}}\,dt, $$

respectively. These functions $\Psi_{P}(s)$ and $\Phi_{P}(s)$ are used below with $P=\frac{n}{\alpha }$.

In [1], Cianchi found the necessary and sufficient conditions for the boundedness of $I_{\alpha }$ on Orlicz spaces.

### Theorem 3.1

[1]

*Let* $0<\alpha <n$. *Let* Φ *and* Ψ *Young functions and let* $\Phi _{n/\alpha}$ *and* $\Psi_{n/\alpha}$ *be the Young functions defined as in* (3.2) *and* (3.1), *respectively*. *Then*

(i) $I_{\alpha }$ *is bounded from* $L^{\Phi}({\mathbb{R}^{n}})$ *to* $WL^{\Psi }({\mathbb{R}^{n}})$ *if and only if*
  3.3 $$ \int_{0}^{1}\widetilde{\Phi}(t)/t^{1+n/(n-\alpha)}\,dt< \infty\quad\textit{and }\Phi_{n/\alpha} \textit{ dominates } \Psi \textit{ globally.} $$
(ii) $I_{\alpha }$ *is bounded from* $L^{\Phi}({\mathbb{R}^{n}})$ *to* $L^{\Psi }({\mathbb{R}^{n}})$ *if and only if*
  3.4 $$ \int_{0}^{1}\widetilde{\Phi}(t)/t^{1+n/(n-\alpha)}\,dt< \infty ,\qquad \int _{0}^{1}\Psi(t)/t^{1+n/(n-\alpha)}\,dt< \infty , $$
  Φ *dominates* $\Psi_{n/\alpha}$ *globally and* $\Phi _{n/\alpha}$ *dominates* Ψ *globally*.

For proving our main results, we need the following estimate.

### Lemma 3.2

*If* $B_{0}:=B(x_{0},r_{0})$, *then* $r_{0}^{\alpha}\leq C I_{\alpha } \chi_{B_{0}}(x)$ *for every* $x\in B_{0}$.

### Proof

If $x,y\in B_{0}$, then $\vert x-y \vert \leq \vert x-x_{0} \vert + \vert y-x_{0} \vert <2r_{0}$. Since $0<\alpha <n$, we get $r_{0}^{\alpha -n}\leq C \vert x-y \vert ^{\alpha -n}$. Therefore

$$\begin{aligned} I_{\alpha } \chi_{B_{0}}(x)= \int_{{\mathbb{R}^{n}}} \chi_{B_{0}}(y) \vert x-y \vert ^{\alpha -n}\,dy = \int_{B_{0}} \vert x-y \vert ^{\alpha -n}\,dy \geq C r_{0}^{\alpha -n} \vert B_{0} \vert = C r_{0}^{\alpha }. \end{aligned}$$

 □

The following theorem gives necessary and sufficient conditions for the boundedness of the operator $I_{\alpha }$ from $L^{\Phi}({\mathbb{R}^{n}})$ to $WL^{\Psi}({\mathbb{R}^{n}})$ and from $L^{\Phi}({\mathbb{R}^{n}})$ to $L^{\Psi}({\mathbb{R}^{n}})$.

### Theorem 3.3

*Let* $0<\alpha <n$ *and* $\Phi, \Psi\in\mathcal{Y}$.

1. *The condition*
  3.5 $$ r^{\alpha}\Phi^{-1} \bigl(r^{-n} \bigr) + \int_{r}^{\infty} \Phi ^{-1} \bigl(t^{-n} \bigr)t^{\alpha}\frac{\,dt}{t} \le C \Psi^{-1} \bigl(r^{-n} \bigr) $$
  *for all* $r>0$, *where* $C>0$ *does not depend on* *r*, *is sufficient for the boundedness of* $I_{\alpha }$ *from* $L^{\Phi}({\mathbb{R}^{n}})$ *to* $WL^{\Psi}({\mathbb{R}^{n}} )$. *Moreover*, *if* $\Phi\in\nabla_{2}$, *the condition* (3.5) *is sufficient for the boundedness of* $I_{\alpha }$ *from* $L^{\Phi}({\mathbb{R}^{n}})$ *to* $L^{\Psi}({\mathbb{R}^{n}})$.
2. *The condition*
  3.6 $$ r^{\alpha}\Phi^{-1} \bigl(r^{-n} \bigr) \le C \Psi^{-1} \bigl(r^{-n} \bigr) $$
  *for all* $r>0$, *where* $C>0$ *does not depend on* *r*, *is necessary for the boundedness of* $I_{\alpha }$ *from* $L^{\Phi}({\mathbb{R}^{n}})$ *to* $WL^{\Psi }({\mathbb{R}^{n}})$ *and from* $L^{\Phi}({\mathbb{R}^{n}})$ *to* $L^{\Psi}({\mathbb{R}^{n}})$.
3. *If the regularity condition*
  3.7 $$ \int_{r}^{\infty} \Phi^{-1} \bigl(t^{-n} \bigr) t^{\alpha}\frac {dt}{t} \le C r^{\alpha} \Phi^{-1} \bigl(r^{-n} \bigr) $$
  *holds for all* $r>0$, *where* $C>0$ *does not depend on* *r*, *then the condition* (3.6) *is necessary and sufficient for the boundedness of* $I_{\alpha }$ *from* $L^{\Phi}({\mathbb{R}^{n}})$ *to* $WL^{\Psi}({\mathbb{R}^{n}})$. *Moreover*, *if* $\Phi\in\nabla _{2}$, *the condition* (3.6) *is necessary and sufficient for the boundedness of* $I_{\alpha }$ *from* $L^{\Phi}({\mathbb{R}^{n}})$ *to* $L^{\Psi }({\mathbb{R}^{n}})$.

### Proof

(1) For an arbitrary ball $B=B(x,r)$ we represent *f* as

$$ f=f_{1}+f_{2}, \qquad f_{1}(y)=f(y) \chi_{B}(y), \qquad f_{2}(y)=f(y)\chi_{{}^{\complement}{B}}(y), $$

and have

$$I_{\alpha }f(x)=I_{\alpha }f_{1}(x)+I_{\alpha }f_{2}(x). $$

For $I_{\alpha }f_{1}(x)$, following Hedberg’s trick, see [8], we obtain $\vert I_{\alpha }f_{1}(x) \vert \lesssim r^{\alpha }Mf(x)$. For $I_{\alpha }f_{2}(x)$ by Lemma 2.6 we have

$$\begin{aligned} \int_{{}^{\complement}{B}}\frac{ \vert f(y) \vert }{ \vert x-y \vert ^{n-\alpha}}\,dy & \thickapprox \int_{{}^{\complement}{B}} \bigl\vert f(y) \bigr\vert \int_{ \vert x-y \vert }^{\infty }\frac {dt}{t^{n+1-\alpha}}\,dy \\ &\thickapprox \int_{r}^{\infty } \int_{r\leq \vert x-y \vert < t} \bigl\vert f(y) \bigr\vert \,dy\frac {dt}{t^{n+1-\alpha}} \\ &\lesssim \int_{r}^{\infty }\Phi^{-1}\bigl( \bigl\vert B(x,t) \bigr\vert ^{-1}\bigr)t^{\alpha -1} \Vert f \Vert _{L^{\Phi }(B(x,t))}\,dt. \end{aligned}$$

Consequently we have

$$ \bigl\vert I_{\alpha }f(x) \bigr\vert \lesssim r^{\alpha }Mf(x)+ \Vert f \Vert _{L^{\Phi}} \int_{r}^{\infty }t^{\alpha }\Phi ^{-1} \bigl(t^{-n}\bigr)\frac{dt}{t}. $$

Thus, by (3.5) we obtain

$$\begin{aligned} \bigl\vert I_{\alpha } f(x) \bigr\vert \lesssim Mf(x)\frac{\Psi^{-1}(r^{-n})}{\Phi ^{-1}(r^{-n})} + \Vert f \Vert _{L^{\Phi}} \Psi^{-1}\bigl(r^{-n} \bigr). \end{aligned}$$

Choose $r>0$ so that $\Phi^{-1}(r^{-n})=\frac{Mf(x)}{C_{0} \Vert f \Vert _{L^{\Phi}}}$. Then

$$\frac{\Psi^{-1}(r^{-n})}{\Phi^{-1}(r^{-n})}=\frac{(\Psi^{-1}\circ \Phi)(\frac{Mf(x)}{C_{0} \Vert f \Vert _{L^{\Phi}}})}{\frac{Mf(x)}{C_{0} \Vert f \Vert _{L^{\Phi}}}}. $$

Therefore, we get

3.8 $$ \bigl\vert I_{\alpha } f(x) \bigr\vert \leq C_{1} \Vert f \Vert _{L^{\Phi}} \bigl(\Psi^{-1}\circ \Phi\bigr) \biggl(\frac{Mf(x)}{C_{0} \Vert f \Vert _{L^{\Phi}}} \biggr). $$

Let $C_{0}$ be as in (2.3). Then by Theorem 2.7,

$$\begin{aligned} \sup_{r>0}\Psi(r) m \biggl(\frac{ \vert I_{\alpha } f(x) \vert }{C_{1} \Vert f \Vert _{L^{\Phi }}},r \biggr)={}&\sup _{r>0}r m \biggl(\Psi \biggl(\frac{ \vert I_{\alpha } f(x) \vert }{C_{1} \Vert f \Vert _{L^{\Phi}}} \biggr),r \biggr) \\ \leq{}&\sup_{r>0}r m \biggl(\Phi \biggl(\frac{M f(x)}{C_{0} \Vert f \Vert _{L^{\Phi }}} \biggr),r \biggr)\\ \leq{}&\sup_{r>0}\Phi(r) m \biggl( \frac{M f(x)}{ \Vert Mf \Vert _{WL^{\Phi}}},r \biggr)\leq1, \end{aligned}$$

*i.e.*

$$\Vert I_{\alpha }f \Vert _{WL^{\Psi}}\lesssim \Vert f \Vert _{L^{\Phi}}. $$

Let $C_{0}$ be as in (2.4). Since $\Phi\in\nabla_{2}$, by Theorem 2.7, we have

$$\int_{{\mathbb{R}^{n}}}\Psi \biggl(\frac{ \vert I_{\alpha } f(x) \vert }{C_{1} \Vert f \Vert _{L^{\Phi }}} \biggr)\,dx\leq \int_{{\mathbb{R}^{n}}}\Phi \biggl(\frac{M f(x)}{C_{0} \Vert f \Vert _{L^{\Phi}}} \biggr)\,dx\leq \int_{{\mathbb{R}^{n}}}\Phi \biggl(\frac{M f(x)}{ \Vert Mf \Vert _{L^{\Phi}}} \biggr)\,dx\leq1, $$

*i.e.*

$$\Vert I_{\alpha }f \Vert _{L^{\Psi}}\lesssim \Vert f \Vert _{L^{\Phi}}. $$

(2) We shall now prove the second part. Let $B_{0}=B(x_{0},r_{0})$ and $x\in B_{0}$. By Lemma 3.2, we have $r_{0}^{\alpha}\leq C I_{\alpha } \chi _{B_{0}}(x)$. Therefore, by Lemma 2.5, we have

$$\begin{aligned} r_{0}^{\alpha}&\lesssim\Psi^{-1}\bigl( \vert B_{0} \vert ^{-1}\bigr) \Vert I_{\alpha } \chi_{B_{0}} \Vert _{WL^{\Psi}(B_{0})} \lesssim\Psi^{-1}\bigl( \vert B_{0} \vert ^{-1}\bigr) \Vert I_{\alpha } \chi _{B_{0}} \Vert _{WL^{\Psi}} \\ &\lesssim\Psi^{-1}\bigl( \vert B_{0} \vert ^{-1}\bigr) \Vert \chi_{B_{0}} \Vert _{L^{\Phi}}\lesssim \frac{\Psi^{-1}(r_{0}^{-n})}{\Phi^{-1}(r_{0}^{-n})} \end{aligned}$$

and

$$\begin{aligned} r_{0}^{\alpha}&\lesssim\Psi^{-1}\bigl( \vert B_{0} \vert ^{-1}\bigr) \Vert I_{\alpha } \chi_{B_{0}} \Vert _{L^{\Psi}(B_{0})} \lesssim\Psi^{-1}\bigl( \vert B_{0} \vert ^{-1}\bigr) \Vert I_{\alpha } \chi_{B_{0}} \Vert _{L^{\Psi}} \\ &\lesssim\Psi^{-1}\bigl( \vert B_{0} \vert ^{-1}\bigr) \Vert \chi_{B_{0}} \Vert _{L^{\Phi}}\lesssim \frac{\Psi^{-1}(r_{0}^{-n})}{\Phi^{-1}(r_{0}^{-n})}. \end{aligned}$$

Since this is true for every $r_{0}>0$, we are done.

(3) The third statement of the theorem follows from the first and second parts of the theorem. □

From Theorems 3.1 and 3.3 we have the following corollary.

### Corollary 3.4

*Let* $0<\alpha <n$, $\Phi, \Psi\in\mathcal{Y}$ *and the regularity condition* (3.7) *holds*, *then*:

(1) *Condition* (3.3) *holds if and only if condition* (3.6) *holds*.

(2) *Moreover*, *if* $\Phi\in\nabla_{2}$, *then condition* (3.4) *holds if and only if* (3.6) *holds*.

The following result is due to Nakai [2].

### Theorem 3.5

[2]

*Let* $0<\alpha <n$ *and* $\Phi, \Psi\in\mathcal{Y}$. *Assume that the conditions* (3.6) *and* (3.7) *hold*. *Then the operator* $I_{\alpha }$ *is bounded from* $L^{\Phi}({\mathbb {R}^{n}})$ *to* $WL^{\Psi}({\mathbb{R}^{n}})$. *Moreover*, *if* $\Phi\in\nabla_{2}$, *then* $I_{\alpha }$ *is bounded from* $L^{\Phi}({\mathbb{R}^{n}})$ *to* $L^{\Psi}({\mathbb{R}^{n}})$.

### Remark 3.6

Note that in Theorem 3.5 Nakai found the sufficient conditions which ensures the boundedness of the operator $I_{\alpha }$ from $L^{\Phi}({\mathbb{R}^{n}})$ to $L^{\Psi}({\mathbb {R}^{n}})$, including weak version. Theorem 3.3 improves Theorem 3.5 by adding the necessity. Theorems 3.1 and 3.3 are different characterizations for the boundedness of the operator $I_{\alpha }$ from $L^{\Phi}({\mathbb{R}^{n}})$ to $L^{\Psi}({\mathbb {R}^{n}})$, including a weak version.

## Maximal commutator in Orlicz spaces

In this section we investigate the boundedness of the maximal commutator $M_{b}$ in Orlicz spaces.

We recall the definition of the space of $\mathrm{BMO}({\mathbb{R}^{n}})$.

### Definition 4.1

Suppose that $f\in L^{1}_{\mathrm{loc}}({\mathbb{R}^{n}})$, let

$$ \Vert f \Vert _{\ast}=\sup_{x\in{\mathbb{R}^{n}}, r>0} \frac{1}{ \vert B(x,r) \vert } \int_{B(x,r)} \bigl\vert f(y)-f_{B(x,r)} \bigr\vert \,dy, $$

where

$$f_{B(x,r)}=\frac{1}{ \vert B(x,r) \vert } \int_{B(x,r)} f(y)\,dy. $$

Define

$$\mathrm{BMO}\bigl({\mathbb{R}^{n}}\bigr)=\bigl\{ f\in L^{1}_{\mathrm{loc}} \bigl({\mathbb{R}^{n}}\bigr) : \Vert f \Vert _{\ast} < \infty\bigr\} . $$

Modulo constants, the space $\mathrm{BMO}({\mathbb{R}^{n}})$ is a Banach space with respect to the norm $\Vert \cdot \Vert _{\ast}$.

Before proving our theorems, we need the following lemmas and theorem.

### Lemma 4.2

[9]

*Let* $b \in \mathrm{BMO}({\mathbb{R}^{n}})$. *Then there is a constant* $C>0$ *such that*

4.1 $$ \vert b_{B(x,r)}-b_{B(x,t)} \vert \le C \Vert b \Vert _{\ast}\ln\frac{t}{r}\quad \textit{for } 0< 2r< t, $$

*where* *C* *is independent of* *b*, *x*, *r*, *and* *t*.

### Lemma 4.3

[10]

*Let* $f\in \mathrm{BMO}({\mathbb{R}^{n}})$ *and* Φ *be a Young function with* $\Phi\in \Delta_{2}$, *then*

4.2 $$ \Vert f \Vert _{\ast}\thickapprox\sup _{x\in{\mathbb{R}^{n}}, r>0}\Phi^{-1} \bigl( \bigl\vert B(x,r) \bigr\vert ^{-1} \bigr) \bigl\Vert f(\cdot)-f_{B(x,r)} \bigr\Vert _{L^{\Phi}(B(x,r))}. $$

### Theorem 4.4

[11]

*Let* $b\in \mathrm{BMO}({\mathbb{R}^{n}})$ *and* $\Phi\in\nabla_{2}\cap\mathcal{Y}$.

*Then the operator* $M_{b}$ *is bounded on* $L^{\Phi}({\mathbb{R}^{n}})$, *and the inequality*

4.3 $$ \Vert M_{b} f \Vert _{L^{\Phi}}\leq C_{0} \Vert b \Vert _{\ast} \Vert f \Vert _{L^{\Phi}} $$

*holds with constant* $C_{0}$ *independent of* *f*.

The following theorem is valid.

### Theorem 4.5

*Let* $b \in \mathrm{BMO}({\mathbb{R}^{n}})$ *and* Φ *be a Young function*. *Then the condition* $\Phi\in\nabla_{2}$ *is necessary for the boundedness of* $M_{b}$ *on* $L^{\Phi}({\mathbb{R}^{n}})$.

### Proof

Assume that (4.3) holds. For the particular symbol $b(x)=\log \vert x \vert \in \mathrm{BMO}({\mathbb{R}^{n}})$ and $f(x)=\chi_{B_{r}}(x)$, (4.3) becomes

4.4 $$\begin{aligned} \Vert M_{b}\chi_{B_{r}} \Vert _{L^{\Phi}}\leq C_{1} \Vert \chi _{B_{r}} \Vert _{L^{\Phi}}, \end{aligned}$$

where $r=(a_{1}uv)^{-1/n}$, $B_{r}=B(0,r)$, $a_{r}= \vert B_{r} \vert $, $u>0$ and $v>1$. By Lemma 2.5 and (2.1), we have

$$\begin{aligned} \Vert \chi_{B_{r}} \Vert _{L^{\Phi}}=\frac{1}{\Phi ^{-1}(1/ \vert B_{r} \vert )}= \frac{1}{\Phi^{-1}(1/(r^{n} \vert B_{1} \vert ))}=\frac{1}{\Phi ^{-1}(uv)}\leq\frac{1}{uv}\widetilde{ \Phi}^{-1}(uv). \end{aligned}$$

On the other hand, if $x\notin B_{r} $ then $B_{r}\subset B(x,2 \vert x \vert ) $ since for $y\in B_{r} $ we have

$$\begin{aligned} \vert x-y \vert \leq \vert x \vert + \vert y \vert \leq \vert x \vert +r\leq2 \vert x \vert . \end{aligned}$$

Also for each $y\in B_{r} $, we have

$$b(x)-b(y)\geq\log\biggl(\frac{ \vert x \vert }{r}\biggr). $$

Therefore

$$\begin{aligned} M_{b}\chi_{B_{r}}(x)\geq\frac{1}{ \vert B(x,2 \vert x \vert ) \vert } \int_{B(x,2 \vert x \vert )\cap B_{r}} \bigl\vert b(x)-b(y) \bigr\vert \,dy\geq \biggl( \frac{r}{2 \vert x \vert } \biggr)^{n} \log\biggl(\frac{ \vert x \vert }{r}\biggr). \end{aligned}$$

Following the ideas of [12], for $g=\widetilde{\Phi }^{-1}(u)\chi_{B_{s}} $ with $s=(a_{1}u)^{-1/n} $ we obtain

$$\begin{aligned} \int_{\mathbb{R}^{n}}\widetilde{\Phi}\bigl( \bigl\vert g(x) \bigr\vert \bigr)\,dx \leq u \vert B_{s} \vert =us^{n} \vert B_{1} \vert =1. \end{aligned}$$

Since the Luxemburg-Nakano norm is equivalent to the Orlicz norm

$$\Vert f \Vert _{\Phi}^{*}:=\sup \biggl\{ \int_{\mathbb {R}^{n}} \bigl\vert f(x)g(x) \bigr\vert \,dx : \Vert g \Vert_{L^{\widetilde{\Phi}}}\leq1 \biggr\} $$

(more precisely, $\Vert f\Vert_{L^{\Phi}}\leq \Vert f \Vert _{\Phi}^{*}\leq2\Vert f\Vert_{L^{\Phi}}$), it follows that

$$\begin{aligned} \Vert M_{b}\chi_{B_{r}} \Vert _{L^{\Phi}}^{\ast}&= \sup \biggl\lbrace \int_{\mathbb{R}^{n}} \bigl\vert M_{b}\chi_{B_{r}}(x)g(x) \bigr\vert \,dx: \int_{\mathbb {R}^{n}}\widetilde{\Phi}\bigl( \bigl\vert g(x) \bigr\vert \bigr)\,dx\leq1 \biggr\rbrace \\ &\geq\widetilde{\Phi}^{-1}(u) \int_{B_{s}}M_{b}\chi_{B_{r}}(x)\,dx\geq \widetilde{\Phi}^{-1}(u) \int_{B_{s}\setminus B_{r}} \biggl( \frac {r}{2 \vert x \vert } \biggr)^{n} \log\biggl(\frac{ \vert x \vert }{r}\biggr)\,dx \\ &=\frac{\widetilde{\Phi}^{-1}(u)}{2^{n}a_{1}uv} \int_{r< \vert x \vert < s}\frac {1}{ \vert x \vert ^{n}}\log\biggl(\frac{ \vert x \vert }{r} \biggr)\,dx \\ &=\frac{\widetilde{\Phi}^{-1}(u)}{2^{n+1}a_{1}uv}na_{1}\biggl(\log\frac {s}{r} \biggr)^{2}=\frac{\widetilde{\Phi}^{-1}(u)}{2^{n+1}nuv}(\log v)^{2}. \end{aligned}$$

Hence (4.4) implies that

$$\begin{aligned} \frac{\widetilde{\Phi}^{-1}(u)}{2^{n+1}nuv}(\log v)^{2}\leq 2C_{1}\frac{1}{uv} \widetilde{\Phi}^{-1}(uv) \end{aligned}$$

for $u>0$ and $v>1$. Thus, taking $v=\exp(\sqrt{nC_{1}}\cdot2^{\frac {n+3}{2}}) $ we obtain $2\widetilde{\Phi}^{-1}(u)\leq \widetilde {\Phi}^{-1}(u\exp(\sqrt{nC_{1}}\cdot2^{\frac{n+3}{2}})) $ for $u>0 $ or $\widetilde{\Phi}(2t)\leq\exp(\sqrt{nC_{1}}\cdot2^{\frac {n+3}{2}})\widetilde{\Phi}(t) $ for every $t>0$, and so Φ̃ satisfies the $\Delta_{2}$ condition. □

By Theorems 4.4 and 4.5 we have the following result.

### Corollary 4.6

*Let* $b \in \mathrm{BMO}({\mathbb{R}^{n}})$ *and* $\Phi\in\mathcal{Y}$. *Then the condition* $\Phi\in\nabla_{2}$ *is necessary and sufficient for the boundedness of* $M_{b}$ *on* $L^{\Phi}({\mathbb{R}^{n}})$.

### Theorem 4.7

$b\in L^{1}_{\mathrm{loc}}({\mathbb{R}^{n}})$ *and* Φ *be a Young function*. *The condition* $b\in \mathrm{BMO}({\mathbb{R}^{n}})$ *is necessary for the boundedness of* $M_{b}$ *on* $L^{\Phi}({\mathbb{R}^{n}})$.

### Proof

Suppose that $M_{b}$ is bounded from $L^{\Phi}({\mathbb{R}^{n}})$ to $L^{\Phi }({\mathbb{R}^{n}})$. Choose any ball $B=B(x,r)$ in ${\mathbb{R}^{n}}$, by (2.1)

$$\begin{aligned} \frac{1}{ \vert B \vert } \int_{B} \bigl\vert b(y)-b_{B} \bigr\vert \,dy & \le\frac{1}{ \vert B \vert } \int_{B} \frac{1}{ \vert B \vert } \int_{B} \bigl\vert b(y)-b(z) \bigr\vert \chi_{B}(z) \,dz \,dy \\ & \le\frac{1}{ \vert B \vert } \int_{B} M_{b} ( \chi_{B} ) (y) \,dy \\ & \le\frac{2}{ \vert B \vert } \bigl\Vert M_{b} ( \chi_{B} ) \bigr\Vert _{L^{\Phi}(B)} \Vert 1 \Vert _{L^{\widetilde{\Phi}}(B)} \\ & \le\frac{2}{ \vert B \vert } \Vert \chi_{B} \Vert _{L^{\Phi}} \Vert \chi_{B} \Vert _{L^{\widetilde{\Phi}}} \leq C. \end{aligned}$$

Thus $b\in \mathrm{BMO}({\mathbb{R}^{n}})$. □

By Theorems 4.4 and 4.7 we have the following result.

### Corollary 4.8

*Let* Φ *be a Young function with* $\Phi\in\nabla_{2}$. *Then the condition* $b \in \mathrm{BMO}({\mathbb{R}^{n}})$ *is necessary and sufficient for the boundedness of* $M_{b}$ *on* $L^{\Phi}({\mathbb{R}^{n}})$.

## Commutators of Riesz potential in Orlicz spaces

In this section we find necessary and sufficient conditions for the boundedness of the commutators of Riesz potential on Orlicz spaces with the help of the previous section.

In [5], Fu *et al.* found the sufficient conditions for the boundedness of the commutator $[b,I_{\alpha }]$ on Orlicz spaces as follows.

### Theorem 5.1

[5]

*Let* $0<\alpha <n$ *and* $b\in \mathrm{BMO}({\mathbb{R}^{n}})$. *Let* Φ *be a Young function and* Ψ *defined*, *via its inverse*, *by setting*, *for all* $t\in(0,\infty )$, $\Psi^{-1}(t):=\Phi^{-1}(t)t^{-\alpha /n}$. *If* $\Phi,\Psi\in\Delta _{2}\cap\nabla_{2}$, *then* $[b,I_{\alpha }]$ *is bounded from* $L^{\Phi }({\mathbb{R}^{n}})$ *to* $L^{\Psi}({\mathbb{R}^{n}})$.

The following lemma is the analogue of the Hedberg trick for $[b, I_{\alpha }]$.

### Lemma 5.2

*If* $0<\alpha<n$ *and* $f, b\in L^{1}_{\mathrm{loc}}({\mathbb{R}^{n}})$, *then for all* $x \in{\mathbb{R}^{n}}$ *and* $r>0$ *we get*

$$ \int_{B(x,r)}\frac{ \vert f(y) \vert }{ \vert x-y \vert ^{n-\alpha }} \bigl\vert b(x)-b(y) \bigr\vert \,dy\lesssim r^{\alpha } M_{b}f(x). $$

### Proof

We have

$$\begin{aligned} & \int_{B(x,r)}\frac{ \vert f(y) \vert }{ \vert x-y \vert ^{n-\alpha }} \bigl\vert b(x)-b(y) \bigr\vert \,dy\\ &\quad = \sum_{j=0}^{\infty} \int_{2^{-j-1}r \le \vert x-y \vert < 2^{-j}r} \frac { \vert f(y) \vert }{ \vert x-y \vert ^{n-\alpha }} \bigl\vert b(x)-b(y) \bigr\vert \,dy \\ & \quad\lesssim\sum_{j=0}^{\infty} \bigl(2^{-j}r\bigr)^{\alpha }\bigl(2^{-j}r \bigr)^{-n} \int _{ \vert x-y \vert < 2^{-j}r} \bigl\vert f(y) \bigr\vert \bigl\vert b(x)-b(y) \bigr\vert \,dy \lesssim r^{\alpha } M_{b}f(x). \end{aligned}$$

 □

### Lemma 5.3

*If* $b\in L^{1}_{\mathrm{loc}}({\mathbb{R}^{n}})$ *and* $B_{0}:=B(x_{0},r_{0})$, *then*

$$r_{0}^{\alpha} \bigl\vert b(x)-b_{B_{0}} \bigr\vert \leq C \vert b,I_{\alpha } \vert \chi_{B_{0}}(x) $$

*for every* $x\in B_{0}$, *where* $b_{B_{0}}=\frac{1}{ \vert B_{0} \vert } \int_{B_{0}} b(y)\,dy$.

### Proof

If $x,y\in B_{0}$, then $\vert x-y \vert \leq \vert x-x_{0} \vert + \vert y-x_{0} \vert <2r_{0}$. Since $0<\alpha <n$, we get $r_{0}^{\alpha -n}\leq C \vert x-y \vert ^{\alpha -n}$. Therefore

$$\begin{aligned} \vert b,I_{\alpha } \vert \chi_{B_{0}}(x)&= \int_{B_{0}} \bigl\vert b(x)-b(y) \bigr\vert \vert x-y \vert ^{\alpha -n}\,dy\geq C r_{0}^{\alpha -n} \int_{B_{0}} \bigl\vert b(x)-b(y) \bigr\vert \,dy \\ &\geq C r_{0}^{\alpha -n} \biggl\vert \int_{B_{0}} \bigl(b(x)-b(y)\bigr)\,dy \biggr\vert = C r_{0}^{\alpha } \bigl\vert b(x)-b_{B_{0}} \bigr\vert . \end{aligned}$$

 □

The following theorem gives necessary and sufficient conditions for the boundedness of the operator $\vert b,I_{\alpha } \vert $ from $L^{\Phi}({\mathbb {R}^{n}})$ to $L^{\Psi}({\mathbb{R}^{n}})$.

### Theorem 5.4

*Let* $0<\alpha <n$, $b\in \mathrm{BMO}({\mathbb{R}^{n}})$ *and* $\Phi,\Psi\in\mathcal{Y}$.

1. *If* $\Phi\in\nabla_{2}$ *and* $\Psi\in\Delta_{2}$, *then the condition*
  5.1 $$ r^{\alpha}\Phi^{-1} \bigl(r^{-n} \bigr) + \int_{r}^{\infty} \biggl(1+\ln\frac{t}{r} \biggr) \Phi^{-1} \bigl(t^{-n} \bigr)t^{\alpha} \frac {\,dt}{t} \le C \Psi^{-1} \bigl(r^{-n} \bigr) $$
  *for all* $r>0$, *where* $C>0$ *does not depend on* *r*, *is sufficient for the boundedness of* $[b,I_{\alpha }]$ *from* $L^{\Phi}({\mathbb{R}^{n}})$ *to* $L^{\Psi }({\mathbb{R}^{n}})$.
2. *If* $\Psi\in\Delta_{2}$, *then the condition* (3.6) *is necessary for the boundedness of* $\vert b,I_{\alpha } \vert $ *from* $L^{\Phi}({\mathbb{R}^{n}})$ *to* $L^{\Psi}({\mathbb{R}^{n}})$.
3. *Let* $\Phi\in\nabla_{2}$ *and* $\Psi\in\Delta_{2}$. *If the condition*
  5.2 $$ \int_{r}^{\infty} \biggl(1+\ln\frac{t}{r} \biggr) \Phi^{-1} \bigl(t^{-n} \bigr) t^{\alpha} \frac{dt}{t} \le C r^{\alpha} \Phi^{-1} \bigl(r^{-n} \bigr) $$
  *holds for all* $r>0$, *where* $C>0$ *does not depend on* *r*, *then the condition* (3.6) *is necessary and sufficient for the boundedness of* $\vert b,I_{\alpha } \vert $ *from* $L^{\Phi}({\mathbb{R}^{n}})$ *to* $L^{\Psi}({\mathbb{R}^{n}})$.

### Proof

(1) For arbitrary $x_{0} \in{\mathbb{R}^{n}}$, set $B=B(x_{0},r)$ for the ball centered at $x_{0}$ and of radius *r*. Write $f=f_{1}+f_{2}$ with $f_{1}=f\chi_{{2B}}$ and $f_{2}=f\chi_{{}^{\complement}{(2B)}}$.

For $x \in B$ we have

$$\begin{aligned} \bigl\vert [b,I_{\alpha}]f_{2}(x) \bigr\vert & \lesssim \int_{\mathbb{R}^{n}} \frac { \vert b(y)-b(x) \vert }{ \vert x-y \vert ^{n-\alpha} } \bigl\vert f_{2}(y) \bigr\vert \,dy \thickapprox \int_{{}^{\complement}{(2B)}} \frac { \vert b(y)-b(x) \vert }{ \vert x_{0}-y \vert ^{n-\alpha} } \bigl\vert f(y) \bigr\vert \,dy \\ &\lesssim \int_{{}^{\complement}{(2B)}} \frac { \vert b(y)-b_{B} \vert }{ \vert x_{0}-y \vert ^{n-\alpha} } \bigl\vert f(y) \bigr\vert \,dy + \int_{{}^{\complement}{(2B)}} \frac { \vert b(x)-b_{B} \vert }{ \vert x_{0}-y \vert ^{n-\alpha} } \bigl\vert f(y) \bigr\vert \,dy =J_{1}+J_{2}(x), \end{aligned}$$

since $x \in B$ and $y\in {}^{\complement}{(2B)}$ implies $\vert x-y \vert \thickapprox \vert x_{0}-y \vert $.

Let us estimate $J_{1}$.

$$\begin{aligned} J_{1}&= \int_{{}^{\complement}{(2B)}}\frac{ \vert b(y)-b_{B} \vert }{ \vert x_{0}-y \vert ^{n-\alpha }} \bigl\vert f(y) \bigr\vert \,dy \thickapprox \int_{{}^{\complement}{(2B)}} \bigl\vert b(y)-b_{B} \bigr\vert \bigl\vert f(y) \bigr\vert \int_{ \vert x_{0}-y \vert }^{\infty}\frac{dt}{t^{n+1-\alpha }}\,dy \\ &\thickapprox \int_{2r}^{\infty} \int_{2r\leq \vert x_{0}-y \vert \leq t} \bigl\vert b(y)-b_{B} \bigr\vert \bigl\vert f(y) \bigr\vert \,dy\frac{dt}{t^{n+1-\alpha }} \lesssim \int_{2r}^{\infty} \int_{B(x_{0},t)} \bigl\vert b(y)-b_{B} \bigr\vert \bigl\vert f(y) \bigr\vert \,dy\frac{dt}{t^{n+1-\alpha }}. \end{aligned}$$

Applying Hölder’s inequality, by (2.1), (4.1), (4.2) and Lemma 2.6 we get

$$\begin{aligned} J_{1} \lesssim{}& \int_{2r}^{\infty} \int_{B(x_{0},t)} \bigl\vert b(y)-b_{B(x_{0},t)} \bigr\vert \bigl\vert f(y) \bigr\vert \,dy\frac{dt}{t^{n+1-\alpha }} \\ &{} + \int_{2r}^{\infty} \vert b_{B(x_{0},r)}-b_{B(x_{0},t)} \vert \int_{B(x_{0},t)} \bigl\vert f(y) \bigr\vert \,dy\frac{dt}{t^{n+1-\alpha }} \\ \lesssim{}& \int_{2r}^{\infty} \bigl\Vert b(\cdot)-b_{B(x_{0},t)} \bigr\Vert _{L^{\widetilde{\Phi }}(B(x_{0},t))} \Vert f \Vert _{L_{\Phi}(B(x_{0},t))} \frac{dt}{t^{n+1-\alpha }} \\ &{} + \int_{2r}^{\infty} \vert b_{B(x_{0},r)}-b_{B(x_{0},t)} \vert \Vert f \Vert _{L_{\Phi}(B(x_{0},t))}\Phi^{-1} \bigl( \bigl\vert B(x_{0},t) \bigr\vert ^{-1} \bigr)\frac {dt}{t^{1-\alpha }} \\ \lesssim {}&\Vert b \Vert _{*} \int_{2r}^{\infty} \biggl(1+\ln\frac{t}{r} \biggr) \Vert f \Vert _{L_{\Phi}(B(x_{0},t))}\Phi^{-1} \bigl( \bigl\vert B(x_{0},t) \bigr\vert ^{-1} \bigr)\frac {dt}{t^{1-\alpha }} \\ \lesssim {}&\Vert b \Vert _{*} \Vert f \Vert _{L^{\Phi}} \int_{2r}^{\infty} \biggl(1+\ln \frac{t}{r} \biggr) \Phi^{-1} \bigl(t^{-n} \bigr)t^{\alpha} \frac{dt}{t}. \end{aligned}$$

A geometric observation shows $2B\subset B(x,3r)$ for all $x \in B$. Using Lemma 5.2, we get

$$\begin{aligned} J_{0}(x)&:= \bigl\vert [b,I_{\alpha}]f_{1}(x) \bigr\vert \lesssim \int_{2B} \frac { \vert b(y)-b(x) \vert }{ \vert x-y \vert ^{n-\alpha} } \bigl\vert f(y) \bigr\vert \,dy \\ &\lesssim \int_{B(x,3r)} \frac{ \vert b(y)-b(x) \vert }{ \vert x-y \vert ^{n-\alpha} } \bigl\vert f(y) \bigr\vert \,dy \lesssim r^{\alpha } M_{b} f(x). \end{aligned}$$

Consequently, we have

$$J_{0}(x)+J_{1} \lesssim \Vert b \Vert _{*}r^{\alpha } M_{b} f(x)+ \Vert b \Vert _{*} \Vert f \Vert _{L^{\Phi }} \int_{2r}^{\infty} \biggl(1+\ln\frac{t}{r} \biggr) \Phi^{-1} \bigl(t^{-n} \bigr)t^{\alpha} \frac{dt}{t}. $$

Thus, by (5.1) we obtain

$$\begin{aligned} J_{0}(x)+J_{1} & \lesssim \Vert b \Vert _{*} \biggl(M_{b} f(x)\frac{\Psi ^{-1}(r^{-n})}{\Phi^{-1}(r^{-n})}+\Psi^{-1} \bigl(r^{-n}\bigr) \Vert f \Vert _{L^{\Phi }} \biggr). \end{aligned}$$

Choose $r>0$ so that $\Phi^{-1}(r^{-n})=\frac{M_{b} f(x)}{C_{0} \Vert b \Vert _{*} \Vert f \Vert _{L^{\Phi}}}$. Then

$$\frac{\Psi^{-1}(r^{-n})}{\Phi^{-1}(r^{-n})}=\frac{(\Psi^{-1}\circ \Phi)(\frac{M_{b} f(x)}{C_{0} \Vert b \Vert _{*} \Vert f \Vert _{L^{\Phi}}})}{\frac{M_{b} f(x)}{C_{0} \Vert b \Vert _{*} \Vert f \Vert _{L^{\Phi}}}}. $$

Therefore, we get

$$J_{0}(x)+J_{1} \leq C_{1} \Vert b \Vert _{*} \Vert f \Vert _{L^{\Phi}}\bigl(\Psi^{-1}\circ \Phi \bigr) \biggl(\frac{M_{b} f(x)}{C_{0} \Vert b \Vert _{*} \Vert f \Vert _{L^{\Phi}}}\biggr). $$

Let $C_{0}$ be as in (4.3). Consequently by Theorem 4.4 we have

$$\int_{B}\Psi \biggl(\frac{J_{0}(x)+J_{1}}{C_{1} \Vert b \Vert _{*} \Vert f \Vert _{L^{\Phi }}} \biggr)\,dx\leq \int_{B}\Phi \biggl(\frac{M_{b} f(x)}{C_{0} \Vert b \Vert _{*} \Vert f \Vert _{L^{\Phi}}} \biggr)\,dx\leq \int_{{\mathbb{R}^{n}}}\Phi \biggl(\frac{M_{b} f(x)}{ \Vert M_{b} f \Vert _{L^{\Phi}}} \biggr)\,dx\leq1, $$

*i.e.*

5.3 $$ \bigl\Vert J_{0}(\cdot)+J_{1} \bigr\Vert _{L^{\Psi}(B)}\lesssim \Vert b \Vert _{*} \Vert f \Vert _{L^{\Phi}}. $$

In order to estimate $J_{2}$, by (4.2), Lemma 2.6 and condition (5.1), we also get

$$\begin{aligned} \Vert J_{2} \Vert _{L^{\Psi}(B)} & = \biggl\Vert \int_{{}^{\complement}{(2B)}} \frac{ \vert b(\cdot)-b_{B} \vert }{ \vert x_{0}-y \vert ^{n-\alpha }} \bigl\vert f(y) \bigr\vert \,dy \biggr\Vert _{L^{\Psi}(B)} \\ & \thickapprox \bigl\Vert b(\cdot)-b_{B} \bigr\Vert _{L^{\Psi}(B)} \int_{{}^{\complement}{(2B)}} \frac{ \vert f(y) \vert }{ \vert x_{0}-y \vert ^{n-\alpha }}\,dy \\ &\lesssim \Vert b \Vert _{*} \frac{1}{\Psi^{-1} ( \vert B \vert ^{-1} )} \int _{{}^{\complement}{(2B)}} \frac{ \vert f(y) \vert }{ \vert x_{0}-y \vert ^{n-\alpha }}\,dy \\ & \thickapprox \Vert b \Vert _{*} \frac{1}{\Psi^{-1} ( \vert B \vert ^{-1} )} \int_{{}^{\complement}{(2B)}} \bigl\vert f(y) \bigr\vert \int_{ \vert x_{0}-y \vert }^{\infty }\frac{dt}{t^{n+1-\alpha}}\,dy \\ &\thickapprox \Vert b \Vert _{*} \frac{1}{\Psi^{-1} ( \vert B \vert ^{-1} )} \int _{2r}^{\infty } \int_{2r\leq \vert x_{0}-y \vert < t} \bigl\vert f(y) \bigr\vert \,dy\frac{dt}{t^{n+1-\alpha}} \\ &\lesssim \Vert b \Vert _{*} \frac{1}{\Psi^{-1} ( \vert B \vert ^{-1} )} \int _{2r}^{\infty } \int_{B(x_{0},t) } \bigl\vert f(y) \bigr\vert \,dy\frac{dt}{t^{n+1-\alpha}} \\ &\lesssim \Vert b \Vert _{*} \frac{1}{\Psi^{-1} ( \vert B \vert ^{-1} )} \int _{2r}^{\infty } \Vert f \Vert _{L^{\Phi}(B(x_{0},t))} \Phi^{-1} \bigl( \bigl\vert B(x_{0},t) \bigr\vert ^{-1} \bigr) t^{\alpha-1} \,dt \\ &\lesssim \Vert b \Vert _{*} \frac{1}{\Psi^{-1} ( \vert B \vert ^{-1} )} \Vert f \Vert _{L^{\Phi}} \int _{2r}^{\infty }t^{\alpha }\Phi^{-1} \bigl(t^{-n} \bigr)\frac{dt}{t} \\ &\lesssim \Vert b \Vert _{*} \Vert f \Vert _{L^{\Phi}}. \end{aligned}$$

Consequently, we have

5.4 $$ \Vert J_{2} \Vert _{L^{\Psi}(B)}\lesssim \Vert b \Vert _{*} \Vert f \Vert _{L^{\Phi}}. $$

Combining (5.3) and (5.4), we get

5.5 $$ \bigl\Vert [b,I_{\alpha }]f \bigr\Vert _{L^{\Psi}(B)} \lesssim \Vert b \Vert _{*} \Vert f \Vert _{L^{\Phi}}. $$

By taking the supremum over *B* in (5.5), we get

$$\bigl\Vert [b,I_{\alpha }]f \bigr\Vert _{L^{\Psi}}\lesssim \Vert b \Vert _{*} \Vert f \Vert _{L^{\Phi}}, $$

since the constants in (5.5) do not depend on $x_{0}$ and *r*.

(2) We shall now prove the second part. Let $B_{0}=B(x_{0},r_{0})$ and $x\in B_{0}$. By Lemma 5.3, we have $r_{0}^{\alpha } \vert b(x)-b_{B_{0}} \vert \leq C \vert b,I_{\alpha } \vert \chi_{B_{0}}(x)$. Therefore, by Lemmas 4.3 and 2.5

$$\begin{aligned} r_{0}^{\alpha}&\lesssim\frac{ \Vert \vert b,I_{\alpha } \vert \chi_{B_{0}} \Vert _{L^{\Psi }(B_{0})}}{ \Vert b(\cdot)-b_{B_{0}} \Vert _{L^{\Psi}(B_{0})}} \lesssim\Psi ^{-1}\bigl( \vert B_{0} \vert ^{-1}\bigr) \bigl\Vert \vert b,I_{\alpha } \vert \chi_{B_{0}} \bigr\Vert _{L^{\Psi}(B_{0})} \\ &\lesssim\Psi^{-1}\bigl( \vert B_{0} \vert ^{-1}\bigr) \bigl\Vert \vert b,I_{\alpha } \vert \chi_{B_{0}} \bigr\Vert _{L^{\Psi}} \lesssim\Psi^{-1}\bigl( \vert B_{0} \vert ^{-1}\bigr) \Vert \chi_{B_{0}} \Vert _{L^{\Phi}}\lesssim \frac{\Psi^{-1}(r_{0}^{-n})}{\Phi^{-1}(r_{0}^{-n})}. \end{aligned}$$

Since this is true for every $r_{0}>0$, we are done.

(3) The third statement of the theorem follows from the first and second parts of the theorem. □

### Remark 5.5

Theorems 5.1 and 5.4 give different sufficient conditions for the boundedness of the operator $[b,I_{\alpha }]$ from $L^{\Phi}({\mathbb {R}^{n}})$ to $L^{\Psi}({\mathbb{R}^{n}})$. But in Theorem 5.4 we also have necessary conditions for the boundedness of the operator $\vert b,I_{\alpha } \vert $ from $L^{\Phi}({\mathbb {R}^{n}})$ to $L^{\Psi}({\mathbb{R}^{n}})$.

The following theorem is valid.

### Theorem 5.6

*Let* $0<\alpha <n$, $b\in L^{1}_{\mathrm{loc}}({\mathbb{R}^{n}})$ *and* $\Phi,\Psi \in\mathcal{Y}$.

1. *If* $\Phi\in\nabla_{2}$, $\Psi\in\Delta_{2}$ *and the condition* (5.1) *holds*, *then the condition* $b\in \mathrm{BMO}({\mathbb{R}^{n}})$ *is sufficient for the boundedness of* $[b,I_{\alpha }]$ *from* $L^{\Phi}({\mathbb{R}^{n}})$ *to* $L^{\Psi }({\mathbb{R}^{n}})$.
2. *If* $\Psi^{-1}(t) \lesssim\Phi^{-1}(t)t^{-\alpha /n}$, *then the condition* $b\in \mathrm{BMO}({\mathbb{R}^{n}})$ *is necessary for the boundedness of* $\vert b,I_{\alpha } \vert $ *from* $L^{\Phi}({\mathbb{R}^{n}})$ *to* $L^{\Psi}({\mathbb{R}^{n}})$.
3. *If* $\Phi\in\nabla_{2}$, $\Psi\in\Delta_{2}$, $\Psi^{-1}(t) \approx\Phi^{-1}(t)t^{-\alpha /n}$ *and the condition* (5.2) *holds*, *then the condition* $b\in \mathrm{BMO}({\mathbb{R}^{n}})$ *is necessary and sufficient for the boundedness of* $\vert b,I_{\alpha } \vert $ *from* $L^{\Phi}({\mathbb{R}^{n}})$ *to* $L^{\Psi}({\mathbb{R}^{n}})$.

### Proof

(1) The first statement of the theorem follows from the first part of Theorem 5.4.

(2) We shall now prove the second part. Choose any ball $B=B(x,r)$ in ${\mathbb{R}^{n}}$, by Lemmas 2.5 and 2.6

$$\begin{aligned} \frac{1}{ \vert B \vert } \int_{B} \bigl\vert b(y)-b_{B} \bigr\vert \,dy & = \frac{1}{ \vert B \vert } \int_{B} \biggl\vert \frac{1}{ \vert B \vert } \int_{B} \bigl(b(y)-b(z)\bigr)\,dz \biggr\vert \,dy \\ & \le\frac{1}{ \vert B \vert ^{1+\frac{\alpha }{n}}} \int_{B} \frac {1}{ \vert B \vert ^{1-\frac{\alpha }{n}}} \int_{B} \bigl\vert b(y)-b(z) \bigr\vert \chi_{B}(z) \,dz \,dy \\ & \le\frac{C}{ \vert B \vert ^{1+\frac{\alpha }{n}}} \int_{B} \int_{B}\frac { \vert b(y)-b(z) \vert }{ \vert y-z \vert ^{n-\alpha }}\chi_{B}(z) \,dz \,dy \\ & \le\frac{C}{ \vert B \vert ^{1+\frac{\alpha }{n}}} \int_{B} \vert b,I_{\alpha } \vert ( \chi _{B} ) (y) \,dy \\ & \le\frac{C}{ \vert B \vert ^{\frac{\alpha }{n}}} \frac{\Psi^{-1}( \vert B \vert ^{-1})}{\Phi ^{-1}( \vert B \vert ^{-1})} \leq C . \end{aligned}$$

Thus $b\in \mathrm{BMO}({\mathbb{R}^{n}})$.

(3) The third statement of the theorem follows from the first and second parts of the theorem. □

## Characterization of Lipschitz spaces via commutators

In this section, as an application of Theorem 3.3 we consider the boundedness of $[b,I_{\alpha }]$ on Orlicz spaces when *b* belongs to the Lipschitz space, by which some new characterizations of the Lipschitz spaces are given. Such a characterization was given in [13] as an application of the boundedness of $M_{b}$ on Lebesgue spaces.

### Definition 6.1

Let $0 < \beta< 1$, we say a function *b* belongs to the Lipschitz space $\dot{\Lambda}_{\beta}({\mathbb{R}^{n}})$ if there exists a constant *C* such that, for all $x, y \in{\mathbb{R}^{n}}$,

$$\bigl\vert b(x)-b(y) \bigr\vert \le C \vert x-y \vert ^{\beta}. $$

The smallest such constant *C* is called the $\dot{\Lambda}_{\beta }({\mathbb{R}^{n}})$ norm of *b* and is denoted by $\Vert b \Vert _{\dot{\Lambda }_{\beta }({\mathbb{R}^{n}})}$.

To prove the theorems, we need auxiliary results. The first one is the following characterization of Lipschitz space, which is due to DeVore and Sharply [14].

### Lemma 6.2

*Let* $0 < \beta< 1$, *we have*

$$\Vert f \Vert _{\dot{\Lambda}_{\beta}({\mathbb{R}^{n}})}\thickapprox\sup_{B} \frac {1}{ \vert B \vert ^{1+\beta/n}} \int_{B} \bigl\vert f(x)-f_{B} \bigr\vert \,dx, $$

*where* $f_{B}=\frac{1}{ \vert B \vert }\int_{B}f(y)\,dy$.

### Lemma 6.3

*Let* $0<\beta<1$, $0< \alpha <n$, $0< \alpha +\beta <n$ *and* $b\in\dot{\Lambda }_{\beta}({\mathbb{R}^{n}})$, *then the following pointwise estimate holds*:

$$\vert b,I_{\alpha } \vert \bigl( \vert f \vert \bigr) (x) \lesssim \Vert b \Vert _{\dot{\Lambda}_{\beta}({\mathbb{R}^{n}} )}I_{\alpha +\beta }\bigl( \vert f \vert \bigr) (x). $$

### Proof

If $b\in\dot{\Lambda}_{\beta}({\mathbb{R}^{n}})$, then

$$\begin{aligned} \vert b,I_{\alpha } \vert \bigl( \vert f \vert \bigr) (x)&= \int_{{\mathbb{R}^{n}}}\frac { \vert b(x)-b(y) \vert }{ \vert x-y \vert ^{n-\alpha }} \bigl\vert f(y) \bigr\vert \,dy \\ & \lesssim \Vert b \Vert _{\dot{\Lambda}_{\beta}({\mathbb{R}^{n}})}I_{\alpha +\beta }\bigl( \vert f \vert \bigr) (x). \end{aligned}$$

 □

The following theorem is valid.

### Theorem 6.4

*Let* $0<\beta<1$, $0< \alpha <n$, $0< \alpha +\beta <n$, $b\in L^{1}_{\mathrm{loc}}({\mathbb{R}^{n}} )$, $\Phi,\Psi\in\mathcal{Y}$.

1. *If* $\Phi\in\nabla_{2}$ *and the conditions*
  6.1 $$\begin{aligned} \int_{t}^{\infty} \Phi^{-1} \bigl(r^{-n} \bigr) r^{\alpha+\beta }\frac {dr}{r} &\le C t^{\alpha+\beta } \Phi^{-1} \bigl(t^{-n} \bigr), \end{aligned}$$
  6.2 $$\begin{aligned} t^{{-}\frac{\alpha+\beta }{n}}\Phi^{-1} (t )&\le C \Psi^{-1} (t ), \end{aligned}$$
  *hold for all* $t>0$, *where* $C>0$ *does not depend on* *t*, *then the condition* $b\in\dot{\Lambda}_{\beta}({\mathbb{R}^{n}})$ *is sufficient for the boundedness of* $[b,I_{\alpha }]$ *from* $L^{\Phi }({\mathbb{R}^{n}} )$ *to* $L^{\Psi}({\mathbb{R}^{n}})$.
2. *If the condition*
  6.3 $$ \Psi^{-1}(t) \leq C \Phi^{-1}(t)t^{-\frac{\alpha+\beta }{n}}, $$
  *holds for all* $t>0$, *where* $C>0$ *does not depend on* *t*, *then the condition* $b\in\dot{\Lambda}_{\beta}({\mathbb{R}^{n}})$ *is necessary for the boundedness of* $\vert b,I_{\alpha } \vert $ *from* $L^{\Phi }({\mathbb{R}^{n}} )$ *to* $L^{\Psi}({\mathbb{R}^{n}})$.
3. *If* $\Phi\in\nabla_{2}$, *condition* (6.1) *holds and* $\Psi^{-1}(t) \thickapprox\Phi^{-1}(t)t^{-\frac{\alpha+\beta }{n}}$, *then the condition* $b\in\dot{\Lambda}_{\beta}({\mathbb{R}^{n}})$ *is necessary and sufficient for the boundedness of* $\vert b,I_{\alpha } \vert $ *from* $L^{\Phi }({\mathbb{R}^{n}} )$ *to* $L^{\Psi}({\mathbb{R}^{n}})$.

### Proof

(1) The first statement of the theorem follows from Theorem 3.3 and Lemma 6.3.

(2) We shall now prove the second part. Suppose that $\Psi^{-1}(t) \lesssim\Phi^{-1}(t)t^{-(\alpha +\beta )/n}$ and $\vert b,I_{\alpha } \vert $ is bounded from $L^{\Phi}({\mathbb{R}^{n}})$ to $L^{\Psi}({\mathbb{R}^{n}} )$. Choose any ball *B* in ${\mathbb{R}^{n}}$, by Lemmas 2.5 and 2.6

$$\begin{aligned} \frac{1}{ \vert B \vert ^{1+\frac{\beta }{n}}} \int_{B} \bigl\vert b(y)-b_{B} \bigr\vert \,dy & = \frac {1}{ \vert B \vert ^{1+\frac{\alpha +\beta }{n}}} \int_{B} \biggl\vert \frac{1}{ \vert B \vert ^{1-\frac{\alpha }{n}}} \int_{B} \bigl(b(y)-b(z)\bigr)\,dz \biggr\vert \,dy \\ & \le\frac{C}{ \vert B \vert ^{1+\frac{\alpha +\beta }{n}}} \int_{B} \int_{B}\frac { \vert b(y)-b(z) \vert }{ \vert y-z \vert ^{n-\alpha }}\chi_{B}(z) \,dz\,dy \\ & \le\frac{C}{ \vert B \vert ^{1+\frac{\alpha +\beta }{n}}} \int_{B} \vert b,I_{\alpha } \vert ( \chi_{B} ) (y) \,dy \\ & \le\frac{C\Psi^{-1}( \vert B \vert ^{-1})}{ \vert B \vert ^{\frac{\alpha +\beta }{n}}} \bigl\Vert \vert b,I_{\alpha } \vert ( \chi_{B} ) \bigr\Vert _{L^{\Psi}(B)} \\ & \le\frac{C}{ \vert B \vert ^{\frac{\alpha +\beta }{n}}} \frac{\Psi ^{-1}( \vert B \vert ^{-1})}{\Phi^{-1}( \vert B \vert ^{-1})} \leq C. \end{aligned}$$

Thus by Lemma 6.2 we get $b\in\dot{\Lambda}_{\beta }({\mathbb{R}^{n}})$.

(3) The third statement of the theorem follows from the first and second parts of the theorem. □

The following theorem is valid.

### Theorem 6.5

*Let* $0<\beta<1$, $0< \alpha <n$, $0< \alpha +\beta <n$, $b\in L^{1}_{\mathrm{loc}}({\mathbb{R}^{n}} )$, $\Phi,\Psi\in\mathcal{Y}$.

1. *If the conditions* (6.1) *and* (6.2) *are satisfied*, *then the condition* $b\in\dot{\Lambda }_{\beta}({\mathbb{R}^{n}})$ *is sufficient for the boundedness of* $[b,I_{\alpha }]$ *from* $L^{\Phi}({\mathbb{R}^{n}})$ *to* $WL^{\Psi}({\mathbb{R}^{n}})$.
2. *If the condition* (6.3) *holds and* $\frac {t^{1+\varepsilon}}{\Psi(t)}$ *is almost decreasing for some* $\varepsilon>0$, *then the condition* $b\in\dot{\Lambda}_{\beta }({\mathbb{R}^{n}})$ *is necessary for the boundedness of* $\vert b,I_{\alpha } \vert $ *from* $L^{\Phi }({\mathbb{R}^{n}} )$ *to* $WL^{\Psi}({\mathbb{R}^{n}})$.
3. *If* $\Psi^{-1}(t) \thickapprox\Phi^{-1}(t)t^{-\frac{\alpha+\beta }{n}}$, *condition* (6.1) *holds and* $\frac {t^{1+\varepsilon}}{\Psi(t)}$ *is almost decreasing for some* $\varepsilon>0$, *then the condition* $b\in\dot{\Lambda}_{\beta}({\mathbb{R}^{n}})$ *is necessary and sufficient for the boundedness of* $\vert b,I_{\alpha } \vert $ *from* $L^{\Phi }({\mathbb{R}^{n}} )$ *to* $WL^{\Psi}({\mathbb{R}^{n}})$.

### Proof

(1) The first statement of the theorem follows from Theorem 3.3 and Lemma 6.3.

(2) For any fixed ball $B_{0}$ such that $x\in B_{0}$ by Lemma 5.3 we have $\vert B_{0} \vert ^{\alpha/n} \vert b(x)-b_{B_{0}} \vert \lesssim \vert b,I_{\alpha } \vert \chi_{B_{0}}(x)$. Thus, together with the boundedness of $\vert b,I_{\alpha } \vert $ from $L^{\Phi }({\mathbb{R}^{n}})$ to $WL^{\Psi}({\mathbb{R}^{n}})$ and Lemma 2.5,

$$\begin{aligned} \bigl\vert \bigl\{ x\in B_{0}: \vert B_{0} \vert ^{\alpha/n} \bigl\vert b(x)-b_{B_{0}}\bigl\vert > \lambda\bigr\} \bigr\vert &\leq\bigl\vert \bigl\{ x\in B_{0}: \vert b,I_{\alpha } \vert \chi_{B_{0}}(x)> \lambda\bigr\} \bigr\vert \\ & \leq\frac{1}{\Psi (\frac{ \lambda}{C \Vert \chi_{B_{0}} \Vert _{L^{\Phi}}} )} = \frac{1}{\Psi (\frac{ \lambda\Phi ^{-1}( \vert B_{0} \vert ^{-1})}{C} )}. \end{aligned}$$

Let $t>0$ be a constant to be determined later, then

$$\begin{aligned} \int_{B_{0}} \bigl\vert b(x)-b_{B_{0}} \bigr\vert \,dx = {}&\vert B_{0} \vert ^{-\alpha/n} \int_{0}^{\infty} \bigl\vert \bigl\{ x\in B_{0}: \bigl\vert b(x)-b_{B_{0}} \bigr\vert > \vert B_{0} \vert ^{-\alpha/n}\lambda\bigr\} \bigr\vert \,d\lambda \\ ={}& \vert B_{0} \vert ^{-\alpha/n} \int_{0}^{t} \bigl|\bigl\{ x\in B_{0}: \bigl\vert b(x)-b_{B_{0}} \bigr\vert > \vert B_{0} \vert ^{-\alpha/n}\lambda\bigr\} \bigr| \,d\lambda \\ &{} + \vert B_{0} \vert ^{-\alpha/n} \int_{t}^{\infty}\bigl\vert \bigl\{ x\in B_{0}: \bigr\vert b(x)-b_{B_{0}}\bigr|> \vert B_{0} \vert ^{-\alpha/n} \lambda\bigr\} \bigr| \,d\lambda \\ \le{}& t \vert B_{0} \vert ^{1-\alpha/n}+ \vert B_{0} \vert ^{-\alpha/n} \int_{t}^{\infty} \frac {1}{\Psi (\frac{ \lambda\Phi^{-1}( \vert B_{0} \vert ^{-1})}{C} )} \,d\lambda \\ \lesssim{}& t \vert B_{0} \vert ^{1-\alpha/n}+ \frac{ \vert B_{0} \vert ^{-\alpha/n}t}{\Psi (\frac{ t \Phi^{-1}( \vert B_{0} \vert ^{-1})}{C} )}, \end{aligned}$$

where we use $\frac{t^{1+\varepsilon}}{\Psi(t)}$ being almost decreasing in the last step.

Set $t=C \vert B_{0} \vert ^{\frac{\alpha+\beta}{n}}$ in the above estimate, we have

$$\int_{B_{0}} \bigl\vert b(x)-b_{B_{0}} \bigr\vert \,dx \lesssim \vert B_{0} \vert ^{1+\beta /n}. $$

Thus by Lemma 6.2 we get $b\in\dot{\Lambda}_{\beta }({\mathbb{R}^{n}})$ since $B_{0}$ is an arbitrary ball in ${\mathbb{R}^{n}}$.

(3) The third statement of the theorem follows from the first and second parts of the theorem. □

## Conclusions

We have obtained necessary and sufficient conditions for the boundedness of the Riesz potential and its commutators on Orlicz spaces. We have also compared our results with the existing results. Lastly, we conclude this paper by remarking that some new characterizations of the Lipschitz spaces have been given as an application of the above-mentioned results.
